# Sex-Specific Differences in Hospital Transfers of Nursing Home Residents: Results from the HOspitalizations and eMERgency Department Visits of Nursing Home Residents (HOMERN) Project

**DOI:** 10.3390/ijerph17113915

**Published:** 2020-06-01

**Authors:** Alexander Maximilian Fassmer, Alexandra Pulst, Guido Schmiemann, Falk Hoffmann

**Affiliations:** 1 Department of Health Services Research, School VI - Medicine and Health Sciences, Carl von Ossietzky University of Oldenburg, Ammerländer Heerstraße 114–118, 26129 Oldenburg, Germany; falk.hoffmann@uni-oldenburg.de; 2 Department for Health Services Research, Institute of Public Health and Nursing Research, University of Bremen, Grazer Straße 4, 28359 Bremen, Germany; a.pulst@uni-bremen.de (A.P.); schmiemann@uni-bremen.de (G.S.); 3 Health Sciences Bremen, University of Bremen, 28359 Bremen, Germany; 4 Department Institute for General Practice, Hannover Medical School, 30625 Hannover, Germany

**Keywords:** nursing home residents, hospitalization, hospital admission, patient transfer, sexes, acute health care, emergency department, emergency medical services

## Abstract

Nursing home (NH) residents are often transferred to hospital (emergency department (ED) visits or hospital admissions) and this occurs more frequently in males. However, respective reasons are rather unclear. We conducted a multicenter prospective study in 14 northwest German NHs with 802 residents in which NH staff recorded anonymized data between March 2018 and July 2019 for each hospital transfer. Measures were analyzed using descriptive statistics and compared between sexes via univariate logistic regression analyses using mixed models with random effects. Eighty-eight planned transfers (53.5% hospital admissions, 46.5% ED visits) occurred as well as 535 unplanned transfers (63.1% hospital admissions, 36.9% ED visits). The two most common causes for unplanned transfers were deteriorations of health status (35.1%) and falls/accidents/injuries (33.5%). Male transferred residents were younger, more often married; their advance directives were more commonly not considered correctly and the NH staff identified more males nearing the end of life than females (52.9% vs. 38.2%). Only 9.2% of transfers were rated avoidable. For advance directive availability and NH staff’s perceptions on transfer conditions, we found marked inter-facility differences. There might be sociocultural factors influencing hospital transfer decisions of male and female nursing home residents and facility characteristics that may affect transfer policy.

## 1. Introduction

Demographic aging affects population structures all over the world. Between 2018 and 2100, the share of people aged 65 years or older in the European Union (EU) will rise from 20% to 31% [1]. Simultaneously, the number of care-dependent persons and residents of nursing homes (NHs) is expected to increase. NH residents are mostly older (≥75 years), are often frail, suffering from chronic conditions, physical and cognitive limitations and they often use multiple medications (polypharmacy) [2,3]. Thus, NH residents have an increased risk for transfers to hospital, which includes emergency department (ED) visits with subsequent discharge to the NH (in the following named ED visits) and hospital admissions [4,5]. Many of these transfers are considered inappropriate [6,7,8,9], particularly at the end of life [10]. Transfer rates differ substantially across different regions, NH populations and time periods [11,12,13], and they have shown to be higher in Germany compared with other Western industrialized countries [12] with 1.2 hospital admissions and 0.5 ED visits per resident and year, respectively [4].

A systematic review showed that in all 20 identified studies assessing the influence of sex on hospitalizations of NH residents, males are more often transferred than females [12]. Recently, we could find a statistically significant risk for males compared with females for both acute hospital admissions (relative risk (RR): 1.23) and ED visits (RR: 1.43) [4]. Another systematic review on end-of-life hospitalizations of NH residents found the same sex differences and, furthermore, that male residents died more often in hospital than females [10]. Surprisingly, the reasons for these findings are rather unclear and only a few of the respective studies discussed them at all [10,12]. Possible explanations could be a more aggressive treatment in males [14,15] and that females are more likely to hold advance directives (ADs) excluding hospital transports [16,17]. The discussion about the appropriateness of hospital transfers of NH residents is ongoing [8,9], and investigating reasons for the illustrated sex differences is essential to reduce inappropriate transfers.

Aim of this study was to investigate sex-specific differences in characteristics of transferred residents and reasons for hospital transfers. Furthermore, we wanted to assess NH staff’s perceptions on the transfer’s benefit for the resident, its avoidability and influencing factors.

## 2. Materials and Methods

### 2.1. Study Design 

This study is part of the HOspitalisations and eMERgency department visits of Nursing home residents (HOMERN) project, which explores the health care of NH residents with a focus on hospital transfers. For this multicenter study, a convenience sample of 14 NHs providing long-term care in northwestern Germany, covering the federal state Bremen and parts of the federal state Lower Saxony, were included. The facilities were heterogeneous regarding their location (urban and rural), size (number of beds) and sponsorship (non-profit and private for-profit). Specialized care facilities (e.g., only for residents with dementia) and care units only providing short-term care were excluded. Participating NHs were required either to include all residents or only residents living in selected care units.

From the 14 NHs, data were collected between March 2018 and July 2019. After formal training in handling of the questionnaire, nursing staff of the NHs (predominantly nursing managers) prospectively recorded information for each hospital transfer (ED visits and hospital admissions, planned and unplanned) for 12 months. Active participation and informed consent of the residents were not required because the study relied solely on data from the existing medical records in the NHs and staff’s perceptions. During the study period, there was continuous and regular telephone and personal contact between the researchers and the NHs.

The study was approved by the ethics committee of the Medical Association in Bremen (RA/RE-613, 16 February 2018) and the data privacy management department of the University of Bremen (6 March 2018). This article follows the “Strengthening the Reporting of Observational Studies in Epidemiology (STROBE)” reporting guideline [18].

### 2.2. Data Collection and Assessed Variables

Data for each hospital transfer were recorded via a paper-based, four-page questionnaire. We assessed characteristics of the resident and the respective transfer, and, if the transfer was unplanned, further information about decision-making and influencing factors. The questionnaire was developed by a multidisciplinary team of health scientists and general practitioners (GPs). Furthermore, insights from interviews of nursing managers/care unit managers, findings of the existing literature [6,7,8,9,19,20,21,22,23] and results from one GPs’ quality circles were taken into account. After a pilot run of one month in three NHs, the nursing staff comments were incorporated into the final version.

For each hospital transfer, the following sociodemographic and health-related data of the respective resident were assessed: year of birth, sex (male/female), marital status (4 groups), dementia diagnosis (yes/no), dementia stage (3 groups), enrollment in specialized outpatient palliative care (SAPV, yes/no) and need of care (5 groups). According to German long-term care insurance, all residents are classified into one of five care grades reflecting the person’s independence and competences considering physical, cognitive or psychological impairments [24]. The resident’s performance in activities of daily living should be assessed using the modified Barthel Index (BI) developed by Shah et al. [23]. The BI sums ten domains (e.g., feeding, toilet use) leading to a total score between 0 (completely dependent) and 100 points (completely independent). For the identification of NH residents nearing the end of life, we used the surprise question (“Would you be surprised if this resident died within the next 6 months?”) [25]. Additionally, the NH staff stated if the resident held an AD and assessed the contained information via an adaption of the emergency advance directive called the patient directive for life sustaining measures (PALMA) [26]. The possible responses were: full clinical emergency treatment (including cardiopulmonary resuscitation), limited clinical treatment (without cardiopulmonary resuscitation and intensive care) and preclinical emergency treatment in the NH.

**Figure 1:**
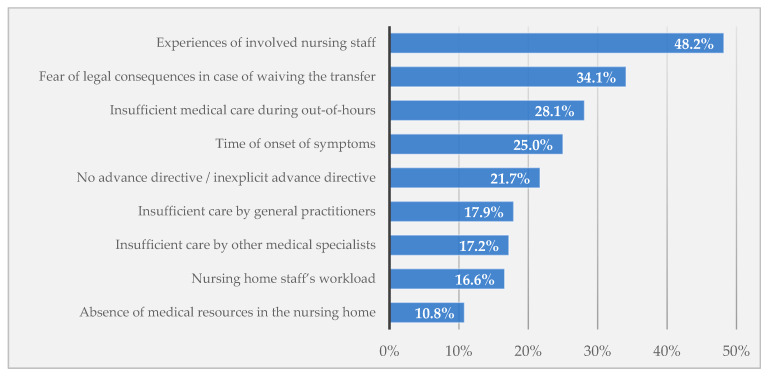
Influencing factors rated as “rather or very important” for unplanned hospital transfers by nursing home staff.

The following information on the respective hospital transfer were assessed: date and time slot of transfer, whether the resident died during transfer or hospitalization, outcome of the transfer (ED/hospital admission), discharge date and reason for transfer (6 groups for planned transfers and 6 groups for unplanned transfers). For unplanned transfers, furthermore, we wanted to know the staff’s perceptions on (a) the transfer’s benefit for the resident, (b) its avoidability and (c) influencing factors of this transfer. Derived from the literature [6,7,8,9,19,20,21,22] and expert meetings, the staff assessed the relevance of nine factors for each case (see Figure 1) on a 5-point Likert scale ranging from ‘0 = no relevance’ to ‘4 = high relevance’. Additionally, each factor could be rated as ‘not assessable’.

### 2.3. Statistical Analyses

All characteristics were compared between sexes using descriptive statistics. We presented categorical data as *n* (%), and for continuous data we stated the mean with standard deviation (SD). Because of missing values, denominators differed between questions. We categorized the resident’s age at time of transfer into four groups (≤69, 70–79, 80–89 and ≥90 years) and combined the two lowest care grades (0 and 1). The BI was grouped into five categories according to the German modification of the International Classification of Diseases, 10th Revision (ICD-10-GM). Analyses were further stratified for planned and unplanned transfers, but our main analysis was on unplanned transfers.

For unplanned hospital transfers, we compared all resident and transfer characteristics by sex, calculating *p*-values derived from univariate logistic regression analyses. These were cluster-adjusted using mixed models with random effects.

The SAS program for Windows, version 9.4 (SAS Institute Inc., Cary, NC, USA) was used for all analyses.

## 3. Results

### 3.1. Characteristics of Transferred Nursing Home Residents

The final sample consisted of 14 NHs (nine with non-profit ownership and five with private-for-profit ownership) with a total of 802 residents (mean: 57.3 residents, 26 to 114 per facility). During the observation time of one year, 626 hospital transfers were carried out (0.78 transfers per resident per year). A total of 535 unplanned transfers (0.67 per resident per year) occurred (63.1% hospital admissions, 36.9% ED visits) as well as 88 planned transfers (0.11 per resident per year; 53.5% hospital admissions, 46.5% ED visits). Three hospital transfers were excluded from further analyses since the residents died during the transfer process and NH staff provided only demographic information on the questionnaires.

Residents that were transferred to hospital unplanned (*n* = 535) were on average 83.8 years old (SD: 9.3) and more often female (70.2%). Almost two-thirds were assigned to care grades 3 or 4 (severe or extremely severe limitations on independence or skills) and more than 40.0% showed severe or total dependency regarding BI (see Table 1). Every second unplanned transferred NH resident (51.3%) had a dementia diagnosis (83.4% of them in a moderate or severe stage). The nursing staff identified 42.5% of unplanned transferred residents near the end of life (surprise question) and only four transferred residents were enrolled in SAPV. For less than half of residents, an AD was available with a range among the 14 participating facilities from 8.3%–80.0%. Two-thirds of these documents (*n* = 162) allowed limited clinical treatment in hospital while a minority of 16.6% allowed only preclinical emergency treatment in the NH.

For some of the characteristics of unplanned transferred residents, we could see conspicuous differences between sexes (see Table 1). Males were likely to be older than females (84.7 vs. 81.7 years) and in higher age groups (*p* = 0.0033) and they were more often married/in a relationship (32.5% vs. 12.0%) and less often widowed (43.5% vs. 72.3%; *p* < 0.0001) than females. Furthermore, males had a higher need of care (*p* = 0.0030) and the NH staff identified more male residents near the end of life than females (52.9% vs. 38.2%; *p* = 0.0052). For the BI group, dementia diagnosis and the availability of ADs, there were no significant differences between males and females. However, males’ ADs allowed more often only preclinical emergency treatment in the NH than females’ directives (29.7% vs. 11.9%; *p* = 0.0269).

In the 88 planned transfers, the NH residents were more often male (52.3%), younger (mean: 77.0 years) and only a fifth had a dementia diagnosis. The most common reasons for these transfers were surgeries (32.2%), catheter changes (24.1%) and scheduled examinations (20.7%). It is striking to note that women underwent surgery more frequently than men and that in almost all catheter changes, the NH residents were male (Table S1).

### 3.2. Description of Unplanned Hospital Transfers

Most NH residents were transferred from Monday to Friday (75.7%). Almost one-quarter of transfers occurred at the weekend (see Table 2). More than 60% of transfers resulted in hospital admissions with a mean length of 8.4 days (SD: 7.8). The two most common reasons for hospital transfers were deteriorations of health status (35.1%) and falls/accidents/injuries (33.5%). Overall, 42 of 334 hospitalized residents died during hospital stay (12.6%).

Weekday of transfer did not differ between sexes. Females were slightly more commonly admitted to hospital than males (65.2% vs. 58.0%). However, males were on average 1.1 days longer hospitalized and they tended to die more often during hospitalization (15.4% vs. 11.6%). For the distribution of transfer reasons, we could see significant differences between sexes (*p* < 0.0001). Falls occurred more often in females (38.1% vs. 22.6% in males) and males had complications more often with the catheter/tube (17.0% vs. 2.9% in females) and psychiatric/neurologic disorders (10.1% vs. 5.6% in females).

### 3.3. Nursing Home Staff’s Perceptions on Unplanned Hospital Transfers

NH staff stated that the resident did not have a benefit from the unplanned transfer in 27.0% (range among NHs: 0–61.1%). Besides, this assessment differed by transfer reason ranging between 8.1% (complications with catheter/tube) and 48.7% (psychiatric/neurologic conditions). Overall, a small proportion of transfers (9.2%) was rated avoidable (range among NHs: 0–37.0%). The influencing factors that were rated most frequently as “rather or very important” for the respective transfer were: experiences of involved nursing staff (48.2%), fear of legal consequences in case of waiving the transfer (34.1%), insufficient medical care during out-of-hours (28.1%), time of onset of symptoms (25.0%) and no ADs/inexplicit ADs (21.7%) (see Figure 1). Again, there were large variations in the ratings among the 14 NHs. However, for these three issues, we could not find any sex-specific differences.

## 4. Discussion

### 4.1. Comparison of Findings with the Literature 

To the best of our knowledge, this is one of the first studies analyzing and comparing characteristics of hospital transfers from NHs by resident’s sex. The two most common causes for unplanned transfers were deteriorations of health status and falls/accidents/injuries. Male unplanned transferred residents were younger and more often married. They frequently had a higher need of care and the NH staff identified more males near the end of life than females. Males’ ADs more commonly allowed only preclinical emergency treatment in the NH. Furthermore, we saw sex-specific differences in the distribution of transfer reasons. While the NH staff’s assessment of the transfer’s benefit, avoidability and influencing factors did not differ by resident’s sex, we could detect marked differences by facility for these three issues.

Almost 13% of hospitalized NH residents died during their hospital stay. End-of-life hospitalizations among NH residents are common [10] and also in our study, the NH staff identified over 40% of transferred residents as nearing the end of their lives. However, not even half of the residents held an AD. Two postal surveys within the HOMERN project asked GPs [27] and NH staff [28] about end-of-life care in German NHs. The survey respondents estimated that 28% (NH staff, [28]) and 40% (GPs, [27]) of residents’ ADs are not taken into account according to their documented care wishes. In our data, this refers to the 40 transferred residents only allowing preclinical emergency treatment in the NH in their ADs. Facing the 313 unplanned transfers with no/not assessable ADs, the concept of advance care planning (ACP) is not comprehensively implemented in German NHs. ACP is a process in which persons discuss and record their care preferences in case of health deterioration [29]. In der Schmitten et al. [30] showed that its implementation in German NHs leads to more ADs.

Among the characteristics of unplanned transfers, there were statistically significant sex-specific differences in the transfer reasons. Similar to Ramroth et al. [31], we found deteriorations of health status and psychiatric/neurologic conditions more commonly among males and falls/accidents/injuries as the most common reason leading to hospital transfer among females. On top of that, a striking difference can be seen for the category complications with catheter/tube, with a substantially larger proportion in males than in females (17.0% vs. 2.9%). These findings might to some extent explain sex-specific differences in hospitalization rates. However, males also die more often in hospital, and other reasons should also be considered. Stall et al. [15] hypothesized that hospital transfer differences between males and females might be more associated with gender (sociocultural factors) than with sex (biological factors) as, for example, women take part more often in ACP and discuss their preferences and wishes more often with their relatives. We could also see that female residents held ADs slightly more often. This was also shown by other studies [16,17]. The finding that male residents receive more invasive and burdensome interventions at the end of life was also shown by Stall et al. [15]. This was also found for other circumstances as, for example, in cancer patients. Male cancer patients receive aggressive and non-beneficial care nearing death more commonly [32], as well as hospitalizations and imaging scans in their last 30 days of life [33]. However, this appears somewhat contradictory since, according to our findings, males wished more often to receive only preclinical treatment in the NH, but these care wishes documented in ADs were disregarded. This needs to be discussed in the context that male NH residents more often still have a partner than females, which are more often widowed. This finding is comparable with the literature [34]. On the one hand, partners can ensure that care wishes are followed. On the other hand, being married still and having a partner who advocates for their care might also potentially lead to more aggressive treatment. This might also be supported by the fact that prognosis of males is somewhat poorer as transferred male residents in our study more often had higher need of care and they were more commonly rated as nearing the end of life than females. There might be some form of unintended biases in decision-making, so called “implicit bias”, that may contribute to differences or inequalities in health care [35], including the decision to transfer NH residents to hospital. Taken together, we believe that other explanations for these sex-specific differences might play a more important role than diagnoses.

Besides resident’s sex, there might be facility-level characteristics predicting hospital transfers [36]. Hence, we could not detect sex-specific differences in the NH staff’s assessments on transfer benefit, avoidability and influencing factors. However, the broad inter-facility ranges in these staff perceptions and the AD availability indicate that managing cases of acute deterioration varies considerably among NHs.

### 4.2. Limitations and Strengths

When interpreting our findings, some limitations and strengths have to be considered. First, the 14 participating facilities willing to take part in our study were a convenience sample in northwestern Germany. Thus, selection bias cannot be ruled out, which may influence the generalizability to all German NHs. However, we tried to recruit a heterogenous sample of NHs with respect to facility sizes, regions (urban and rural) and sponsorships (non-profit and private-for-profit). Nevertheless, the annual (unplanned) transfer rate we calculated was considerably lower than in a former study conducted with German administrative data (1.7 per resident per year, [4]). A possible reason for this may be better health status of the NH residents in our convenience sample compared to the total German institutionalized population. Besides, it might be that the NH staff did not report all hospital transfers during the study period (under-reporting). Indeed, two members of the research team regularly contacted the NHs and the facilities received an expense allowance for each questionnaire. Although most of the requested information should have been assessed by the NH staff relying on existing medical records, a recall bias is a further possible limitation. However, in contrast to studies based on administrative data, we had information with respect to marital status, BI and wishes for end-of-life care. Furthermore, we could assess the NH staff’s rating of influencing factors, benefit and avoidability of transfers. Since nursing staff provide the bulk of care for NH residents [21], they are one main group for assessing the complexity around hospital transfers from NH residents [7,8,9,21]. When rating influencing factors and avoidability of transfers, a social desirability bias can occur, i.e., nursing staff may present themselves or their facility more favorably. However, we tried to prevent this kind of response as effectively as possible, e.g., by emphasizing the anonymity of data analysis and the pooled presentation of findings. Still, we saw much lower ratings of transfer avoidability than in most previous research [6,8,9]. Finally, comparability of our findings with the international literature is limited by heterogeneity in terms of institutionalized populations, health care systems and observational periods [11,12].

## 5. Conclusions

NH residents are frequently transferred to hospital and males are at an increased transfer risk. The current sex-stratified findings provide deeper insights on the management of NH residents with acute situations. ADs were available for less than half of transferred residents only. A better practical implementation of ACP could increase the availability of valid ADs so that residents’ care wishes can be respected. We could see some differences between males and females. Male transferred residents had a higher need of care and they were more likely to be end-of-life patients, making their medical prognosis poorer compared to females. Further research is needed to confirm and explain our finding of males’ disregarded care wishes. The influence of family members on transfer decision has to be examined. Furthermore, there might be an association between the hospital transfer policy and NH characteristics. Our findings underline the importance of sex- and facility-stratified analyses for research on health care in NHs.

## Figures and Tables

**Table 1:** Characteristics of unplanned transferred nursing home residents.

	Total Transfers (*n* = 535)		Transfers of Females (*n* = 375)		Transfers of Males (*n* = 159)		*p*-Value
Age of the residents at time of hospital transfer (years) *							
Mean (SD)	83.8	(9.3)	84.7	(8.8)	81.7	(10.1)	
≤69	42	(7.9%)	22	(5.9%)	20	(12.7)	0.0033
70–79	95	(17.9)	56	(15.1)	38	(24.1)	
80–89	252	(47.5)	188	(50.5)	64	(40.5)	
≥90	142	(26.7)	106	(28.5)	36	(22.8)	
Marital status of the residents *							
Single	47	(9.0%)	32	(8.7%)	15	(9.7%)	<0.0001
Married/in a relationship	95	(18.2)	44	(12.0%	50	(32.5)	
Divorced/permanently separated	48	(9.2%)	26	(7.1%)	22	(14.3)	
Widowed	333	(63.7)	266	(72.3)	67	(43.5)	
Care grade of the residents *							
1 or 2 (few or significant limitations on independence or skills)	108	(20.6)	88	(23.9)	20	(12.9)	0.0030
3 (severe limitations on independence or skills)	173	(33.0)	121	(32.9)	52	(33.6)	
4 (extremely severe limitations on independence or skills)	167	(31.9)	101	(27.5)	65	(41.9)	
5 (extremely severe limitations on independence or skills with special demands on care provision)	76	(14.5)	58	(15.8)	18	(11.6)	
Dementia diagnosis of the residents *							
No	259	(48.7)	178	(47.6)	80	(51.0)	0.4969
Yes	273	(51.3)	196	(52.4)	77	(49.0)	
Stage: mild	44	(16.6)	31	(16.4)	13	(17.1)	0.0529
Stage: moderate	124	(46.8)	97	(51.3)	27	(35.5)	
Stage: severe	97	(36.6)	61	(32.3)	36	(47.4)	
Barthel Index: residents’ activities of daily living (points, ICD-10-GM) *							
Mean (SD)	43.4	(24.9)	44.6	(24.5)	40.9	(25.5)	
80–100: U50.0/1 (slight/no dependency)	43	(8.3%)	29	(8.0%)	14	(9.2%)	0.1356
60–75: U50.2 (mild dependency)	123	(23.8)	98	(27.0)	25	(16.3)	
40–55: U50.3 (moderate dependency)	141	(27.3)	97	(26.7)	44	(28.8)	
20–35: U50.4 (severe dependency)	105	(20.3)	70	(19.3)	35	(22.9)	
0–15: U50.5 (total dependency)	105	(20.3)	69	(19.0)	35	(22.9)	
Resident’s wish for end-of-life care *							
Unknown	282	(53.1)	191	(51.3)	92	(58.2)	0.2469
Advance directive available	249	(46.9)	181	(48.7)	66	(41.8)	
Full clinical emergency treatment	9	(3.7%)	9	(5.1%)	0	(0.0%)	0.0338
Limited clinical treatment	162	(66.9)	120	(67.8)	41	(64.1)	
Preclinical emergency treatment in the NH	41	(16.9)	22	(12.4)	19	(29.7)	
Assessment not possible	30	(12.4)	26	(14.7)	4	(6.3%)	
Surprise question (estimating 6-month mortality) *							
Likely	222	(42.5)	140	(38.2)	82	(52.9)	0.0052
Unlikely	301	(57.6)	227	(61.9)	73	(47.1)	

SD: Standard deviation; ICD-10-GM: International Classification of Diseases, 10th version, German modification; NH: Nursing home. * Numbers differ due to missing values.

**Table 2:** Characteristics of unplanned hospital transfers.

	Total Transfers (*n* = 535)		Transfers of Females (*n* = 375)		Transfers of Males (*n* = 159)		*p*-Value
Weekday of the hospital transfer *							
Monday to Friday	404	(75.7%)	283	(75.5%)	120	(76.0%)	0.9649
Saturday and Sunday	130	(24.3%)	92	(24.5%)	38	(24.1%)	
Outcome of the hospital transfer *							
ED visit with discharge to the NH	195	(36.9%)	129	(34.8%)	66	(42.0%)	0.0937
Hospital admission	334	(63.1%)	242	(65.2%)	91	(58.0%)	
Died during hospitalization	42	(12.6%)	28	(11.6%)	14	(15.4%)	0.3505
Length of hospitalization (days) *^,#^							
Mean (SD)	8.4	(7.8)	8.1	(7.0)	9.2	(9.7)	
1–4	95	(31.4%)	71	(32.3%)	23	(28.1%)	0.8045
5–9	123	(40.6%)	89	(40.5%)	34	(41.5%)	
10+	85	(28.1%)	60	(27.3%)	25	(30.5%)	
Reason for hospital transfer *							
Deterioration of health status (e.g., fever, infection, dyspnea, exsiccosis)	188	(35.1%)	128	(34.1%)	60	(37.7%)	<0.0001
Fall, accident, injury	179	(33.5%)	143	(38.1%)	36	(22.6%)	
Psychiatric/neurologic conditions (e.g., challenging behavior, stroke)	38	(7.1%)	21	(5.6%)	16	(10.1%)	
Complications with catheter/tube (e.g., blood in urine (hematuria))	38	(7.1%)	11	(2.9%)	27	(17.0%)	
Pain, not fall-induced	33	(6.2%)	27	(7.2%)	6	(3.8%)	
Others (e.g., bleedings, gastrointestinal symptoms)	59	(11.0%)	45	(12.0%)	14	(8.8%)	

ED: Emergency department; NH: Nursing home; SD: Standard deviation. * Numbers differ due to missing values. ^#^ Only for hospital admissions.

## Supplementary Materials

The following are available online at https://www.mdpi.com/1660-4601/17/11/3915/s1, Table S1: Characteristics of planned transferred nursing home residents and the respective hospital transfers.

## Abbreviations:

ACP	advance care planning
BI	Barthel Index
ED	emergency department
GP	general practitioner
NH	nursing home
ICD-10-GM	German modification of the International Classification of Diseases, 10th Revision
PALMA	patient directive for life sustaining measures
SAPV	specialized outpatient palliative care
