# Supplementary material for: Sex-Specific Differences in Hospital Transfers of Nursing Home Residents: Results from the HOspitalizations and eMERgency Department Visits of Nursing Home Residents (HOMERN) Project
Source: Int J Environ Res Public Health. 2020 Jun 1;17(11):3915. doi: 10.3390/ijerph17113915 (PMC7312075; doi:10.3390/ijerph17113915)
Supplement: Supplementary file 1 [file ijerph-17-03915-s001.pdf]

Table S1. Characteristics of planned transferred nursing home residents and the respective hospital transfers.

	**Total Transfers (*n* = 88)**		**Transfers of Females (*n* = 42)**		**Transfers of Males (*n* = 46)**	
**Age of the residents at time of hospital transfer (years) ***						
Mean (SD)	77.0	(9.4)	78.1	(10.2)	76.0	(8.5)
≤ 69	21	(23.9%)	10	(23.8%)	11	(23.9%)
70–79	32	(36.4%)	12	(28.6%)	20	(43.4%)
80–89	28	(31.8%)	17	(40.5%)	11	(23.9%)
≥ 90	7	(8.0%)	3	(7.1%)	4	(8.7%)
**Marital status of the residents ***						
Single	13	(14.8%)	6	(14.3%)	7	(15.2%)
Married/in a relationship	23	(26.1%)	3	(7.1%)	20	(43.5%)
Divorced/permanently separated	9	(10.2%)	4	(9.5%)	5	(10.9%)
Widowed	43	(48.9%)	29	(69.1%)	14	(30.4%)
**Care grade of the residents ***						
1 or 2 (few or significant limitations on independence or skills)	26	(30.2%)	15	(36.6%)	11	(24.4%)
3 (severe limitations on independence or skills)	23	(26.7%)	16	(39.0%)	7	(15.6%)
4 (extremely severe limitations on independence or skills)	30	(34.9%)	4	(9.8%)	26	(57.8%)
5 (extremely severe limitations on independence or skills with special demands on care provision)	7	(8.1%)	6	(14.6%)	1	(2.2%)
**Dementia diagnosis of the residents ***						
No	71	(80.7%)	30	(71.4%)	41	(89.1%)
Yes	17	(19.3%)	12	(28.6%)	5	(10.9%)
Stage: mild	2	(12.5%)	1	(9.1%)	1	(20.0%)
Stage: moderate	11	(68.8%)	9	(81.8%)	2	(40.0%)
Stage: severe	3	(18.8%)	1	(9.1%)	2	(40.0%)
**Barthel Index: residents' activities of daily living (points, ICD-10-GM) ***						
Mean (SD)	47.8	(26.9)	53.5	(30.2)	42.7	(22.6)
80-100: U50.0/1 (slight/no dependency)	12	(14.0%)	10	(24.4%)	2	(4.4%)
60-75: U50.2 (mild dependency)	24	(27.9%)	11	(26.8%)	13	(28.9%)
40-55: U50.3 (moderate dependency)	14	(16.3%)	6	(14.6%)	8	(17.8%)
20-35: U50.4 (severe dependency)	29	(33.7%)	8	(19.5%)	21	(46.7%)
0-15: U50.5 (total dependency)	7	(8.1%)	6	(14.6%)	1	(2.2%)
**Residents' wish for end-of-life care ***						
Unknown	59	(68.6%)	23	(56.1%)	36	(80.0%)
Advance directive available	27	(31.4%)	18	(43.9%)	9	(20.0%)
Full clinical emergency treatment	0	(0.0%)	0	(0.0%)	0	(0.0%)
Limited clinical treatment	16	(64.0%)	8	(50.0%)	8	(88.9%)

Preclinical emergency treatment in the NH	1	(4.0%)	1	(6.3%)	0	(0.0%)
Assessment not possible	8	(32.0%)	7	(43.8%)	1	(11.1%)
**Surprise question (estimating 6-month mortality) ***						
Likely	13	(14.9%)	5	(12.2%)	8	(17.4%)
Unlikely	74	(85.1%)	36	(87.8%)	38	(82.6%)
**Weekday of the hospital transfer ***						
Monday to Friday	83	(94.3%)	41	(97.6%)	42	(91.3%)
Saturday & Sunday	5	(5.7%)	1	(2.4%)	4	(8.7%)
**Outcome of the hospital transfer ***						
ED visit with discharge to the NH	40	(46.5%)	12	(29.3%)	28	(62.2%)
Hospital admission	46	(53.5%)	29	(70.7%)	17	(37.8%)
**Length of hospital stay [days] *#**						
Mean (SD)	7.4	(9.8)	7.6	(10.8)	6.9	(8.0)
1 – 4	25	(56.8%)	15	(53.6%)	10	(62.5%)
5 – 9	12	(27.3%)	9	(32.1%)	3	(18.8%)
10 +	7	(15.9%)	4	(14.3%)	3	(18.8%)
**Reason for hospital transfer**						
Surgery	28	(32.2%)	18	(42.9%)	10	(22.2%)
Catheter change	21	(24.1%)	1	(2.4%)	20	(44.4%)
Scheduled examination (e.g., X-ray, colonoscopy)	18	(20.7%)	10	(23.8%)	8	(17.8%)
Scheduled therapeutic intervention (e.g., chemotherapy, dialysis, pain therapy)	9	(10.3%)	4	(9.5%)	5	(11.1%)
Wound treatment and check-up (e.g., bandage)	4	(4.6%)	4	(9.5%)	0	(0.0%)
Other	7	(8.1%)	5	(11.9%)	2	(4.4%)

SD: standard deviation; ICD-10-GM: International Classification of Diseases, 10th version, German Modification; ED: emergency department; NH: nursing home; *Numbers differ due to missing values; #Only for hospital admissions
